# The role of globalization in drug development and access to orphan drugs: orphan drug legislation in the US/EU and in Latin America

**DOI:** 10.12688/f1000research.4268.1

**Published:** 2015-02-27

**Authors:** Renée J.G. Arnold, Lida Bighash, Alejandro Bryón Nieto, Gabriela Tannus Branco de Araújo, Juan Gabriel Gay-Molina, Federico Augustovski

**Affiliations:** 1 Department of Preventive Medicine, Mount Sinai School of Medicine, New York, NY, 10029-6574, USA; 2 Arnold Consultancy & Technology, New York, NY, 10023-3458, USA; 3 Quorum Consulting, Inc., San Francisco, CA, 94104, USA; 4 Skaggs School of Pharmacy and Pharmaceutical Sciences, University of Colorado Anschutz Medical Campus, Aurora, CO, 80045, USA; 5 HEORT, Bogotá, Colombia; 6 AxiaBio Life Sciences, São Paulo City, Brazil; 7 T.I. Salud, Col. Juárez, 06600, Mexico; 8 Institute for Clinical Effectiveness and Health Policy & Professor of Public Health, Universidad de Buenos Aires, Ciudad de Buenos Aires, C1414CPT, Argentina

**Keywords:** market access, orphan drugs, Latin America, rare disease

## Abstract

Compared to a decade ago, nearly three times as many drugs for rare diseases are slated for development. This article addresses the market access issues associated with orphan drug status in Europe and the United States in contrast to the legislation in five Latin American (LA) countries that have made strides in this regard--Mexico, Brazil, Colombia, Chile and Argentina. Based on the success of orphan drug legislation in the EU and US, LA countries should strive to adopt similar strategies with regard to rare diseases and drug development. With the implementation of new targeted regulations, reimbursement strategies, and drug approvals, accessibility to treatment will be improved for people afflicted with rare diseases in these developing countries.

## Introduction

Medications for the approximately 7,000 rare diseases in the world account for less than 10% of global pharmaceutical spending. Although significant, the contribution is small compared to pharmaceuticals for common diseases. The definition of a rare disease varies, but it is generally said to affect <1 in 2000 people in the European Union or <200,000 people in the United States ( Acta Pediatra). Given the low incidence and prevalence of these diseases, they individually reach only a small percentage of the global population; together, however, they affect between 6% and 8% (or 420 million to 560 million people), thus imposing a significant global burden. Of this total, approximately 6 million patients affected by rare diseases are in Mexico, 13 million in Brazil, and 3 million in Argentina. México, Argentina and Colombia use the EU definition of rare diseases, while Brazil, Péru and Chile have bills under consideration, but still have not defined rare diseases or orphan drugs.

Orphanet defines an orphan drug as a “drug not developed by the pharmaceutical industry for economic reasons but which responds to public health need”. This includes products to treat rare diseases as well as products withdrawn from the market for economic/therapeutic reasons, e.g., thalidomide, and products that have not yet been developed ^1^. To further complicate matters, certain drugs may be designated as “orphan” only in subpopulations (e.g., the elderly) within a particular non-rare disease indication, such as cancer. Or, a single drug may be developed for a rare disease, but then further developed for non-rare variants of that therapeutic category. Sometimes this last designation is taken even further in that a non-rare disease can be "aggressively" segmented into multiple rare diseases to achieve an orphan designation for a drug ^2^. The lack of clear regulations surrounding orphan drugs in Latin America (LA) is concerning, given the millions of Latin Americans who have heterogeneous access to treatment for their rare diseases. Although the recognition of the special status of rare drugs and diseases is generally regarded as an accepted, positive label that will result in improved health for people in need of care, the controversy lies with exactly how people with orphan diseases in the US and EU, as well as in LA, should be covered in terms of legislation, drug approvals, reimbursement, and investment, especially given the disparate incentives given to the drug industry for the development of orphan drugs.

Approximately 80% of rare diseases have a genetic origin ^3^. The remainder are the result of bacterial and viral infections, allergies or degenerative conditions ^4^. Most rare diseases (75%) are manifested early in life and affect children from 0 to 5 years of age ^5^. They also contribute significantly to morbidity and mortality in the first 18 years of life. The primary challenge is to balance patient demand with the rising costs in the drug development industry due to scientific and technological advancements.

## Organizations

Several organizations have been involved in the development of legislation and advocacy for rare disease research, in addition to being advocates for patients with rare diseases. Some of these organizations are discussed below:

- Orphanet was established in France in 1997 with the support of the French Ministry of Health; its current membership includes 35 countries. Orpha.net is a reference portal for information about orphan drugs and rare diseases. The Orphanet annual report for 2012 states that “Negotiations were initiated with Argentina, Brazil, China, Chile, Japan and Russia” for the purpose of joining the organization. Interestingly, Mexico and Brazil are among the top 10 countries to access data from the orpha.net website ^1^.
- The International Rare Diseases Research Consortium ( IRDiRC) was launched in April 2011 by the European Commission and the US National Institutes of Health to foster international collaboration in rare diseases research. IRDiRC brings researchers and organizations investing in rare disease research together in order to achieve two main objectives—to deliver 200 new therapies for rare diseases, and to develop the means to diagnose most rare diseases by the year 2020 ( IRDiRC).
- Eurordis is a non-governmental alliance of patient organizations representing 590 rare disease patient organizations in 54 countries ^3^.
- The International Conference on Rare Diseases and Orphan Drugs ( ICORD) is a not-for-profit group comprised of academics, patient advocacy groups, regulatory authorities, public policy professionals, and people from the healthcare services industry. The group is primarily involved in policy advocacy. In 2010, a conference convened in Buenos Aires calling for globalization of research on rare diseases and drug development ^6, 7^. The most recent conference took place in Ede, The Netherlands, with the main theme of “The Societal Value of Prevention, Diagnosis, and Treatments of Rare Diseases ( ICORD).
- The European Commission Expert Group on Rare Diseases (previously EUCERD, the European Union Committee of Experts on Rare Diseases) has the mandate to aid the "European Commission with the preparation and implementation of community activities in the field of rare diseases, in cooperation and consultation with the specialized bodies in Member States, the relevant European authorities in the fields of research and public health action and other relevant stakeholders acting in the field" ( EUCERD).

## Legislation

**Figure 1.**
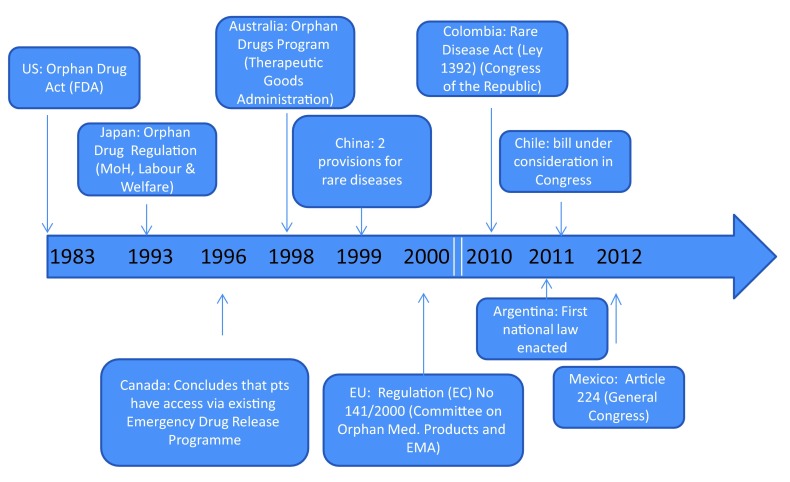
Timeline of legislation surrounding rare diseases in different countries.

As shown in Figure 1, Latin American countries have come relatively "late to the game" in terms of legislation surrounding rare diseases. The state of the legislation will be addressed below and grouped according to the presence or absence of a definition for rare diseases in each country.

### Countries using the EU definition of rare diseases (México, Argentina, Chile and Colombia)

***México.*** A change to a general health law in Mexico was enacted at the beginning of 2012, Article 224; the law was amended to recognize both orphan diseases and the orphan drugs to treat them ^8^. The Mexican Pharmacopeia previously provided a definition of an orphan drug, but it is now officially a Federal Law. However, the new regulations do not provide an “exhaustive body of law” for orphan drugs, and there is no regulation for “exclusivity”.

Furthermore, Seguro Popular is a public health insurance policy initiated in 2003 that expands the accessibility of comprehensive health care services to millions of previously uninsured Mexicans. As of 2011, four rare diseases had been incorporated into the Seguro Popular scheme and seven drugs had been incorporated into the Basic Formulary ( COFEPRIS). Additionally, seven new drugs received the “Orphan Drug Commercialization License” in 2013 and a total of eight rare diseases are now incorporated in the scheme.

Of note, in September 2014 a patient suffering from paroxysmal nocturnal hemoglobinuria filed a lawsuit against the Instituto Mexicano del Seguro Social (Mexican Social Security Institute) or IMSS, claiming that IMSS had the constitutional responsibility to pay for his treatment (eculizumab). However, the court ruled in favor of IMSS, saying that they were only obliged to pay for the treatment if the drug was already included in the Basic Formulary but emphasized that the hospital could submit a request to the General Health Council for the evaluation of the inclusion of eculizumab in the Basic Formulary ( Comunicados de Prensa). This case illustrates that the Basic Formulary defines what can and cannot be acquired by the public healthcare institutions; unfortunately, certain treatments for rare diseases are still not included in the formulary. Thus, there remains a need for regulatory incentives that promote investment for research and development of orphan drugs.

***Argentina.*** Argentina enacted its first national law (Law 26.689) for the healthcare of people with rare diseases in June 2011, largely as a result of advocacy led by the Geiser Foundation (Grupo de Enlace, Investigación y Soporte - Enfermedades Rares), a regional initiative created in 2001 to pool rare disease resources. The law defines rare diseases using the EU definition and requires the health system and public/private social security schemes to provide patient support. The law also mandates the establishment of a national patient registry for neonatal screening programs and the creation of a central coordinating committee ^9, 10^. Although the law was enacted in 2011, it is still not regulated; thus, the law provides a general framework for progression in the arena of rare diseases but has little enforcement power in practice. In 2012 the drug and food regulatory agency (ANMAT) created a commission for the evaluation and authorization of drugs for special conditions, using the same definition as the aforementioned law. Among other mandates, the decree requires intensive post-marketing surveillance for drug approval ( decree 4622/2012).

In 2014 the superintendent of health, the body that regulates the provision of health technologies for the social security and private sectors as well as the reimbursement of selected technologies, created a mentoring system ( Sistema de Tutelaje de Tecnologias Sanitarias Emergentes) of conditional reimbursement where information about patient-relevant outcomes is required for reimbursement ( Ministerio de Salud). Though not specific for orphan status, some rare drugs and diseases are included (i.e., galsulfase for type IV mucopolysaccaridosis and eculizumab for nocturnal paroxysmal hemoglobinuria). Additionally, there is an ongoing legislation project for the creation of a specific fund for rare/catastrophic diseases. Similar to other countries in the region where health is considered a universal right, many of the coverage decisions are enacted through litigation in the judicial system ^11^.

***Chile.*** After 15 years of effort, Chile finally passed a law pertaining to the financing of medications for rare diseases on January 15, 2015 ( La Ley Ricarte Soto). Specifically, the proposal seeks to find a sustainable approach to healthcare coverage for 20 million patients who are affected by rare, costly diseases. Over four years, the government will carry out this grand initiative with an investment of 200 billion pesos. This is 10 times greater than the amount of money that is currently dedicated to such coverage.

Additionally, with the support of the Chile Regional Chapter of the International Society for Pharmacoeconomics and Outcomes Research ( ISPOR), the HTAnetLatAm 2014 Roundtable took place at the Chile Institute of Public Health—Ministry of Health in Santiago, Chile in September of 2014 ( HTAnetLatAm). The roundtable discussion focused on high-cost drugs for rare diseases in LA.

***Colombia.*** In 2010, Colombia passed the Orphan Disease Law and hosted the 2 ^nd^ National Forum of Orphan Diseases ^9^. Specifically, the 2010 Law 1392 recognizes orphan diseases as a significant issue in healthcare due to their low prevalence and high cost to the health system ^12^. As a result, the government has enacted rules to ensure social protection for people with rare diseases. Additionally, through the 1438 Law adopted in 2011, the government confirmed that a rare disease is labeled as an “orphan disease” if the disease is chronically debilitating, life-threatening, and its prevalence is less than 1 in every 5,000 people ^13^. Stemming from these laws, the government made significant regulatory actions within the healthcare information system ^14^. Furthermore, in 2013 the Ministry of Health, through the Colombian Fund for High Cost Diseases (High Cost Account), conducted the first census of patients with orphan diseases, in which 13,168 cases were reported ( Cuenta de Alto Costo). Currently, workshops are being conducted to define models of care in these diseases to ensure adequate access to healthcare that is especially required for this population.

### Countries with their own definition of rare diseases (Brazil)

***Brazil.*** A review of health litigation in Latin American countries revealed that, in Brazil, fewer lawsuits are filed against drugs in the Exceptional Circumstance Drug Dispensing Program (13–31%) as compared to basic medications in the Unified Health System (SUS) (50%), suggesting that a foundation for a solid legal framework for drugs for orphan diseases may already be in the works ( Rev Panam Salud Publica).

In 2013 a public consultation took place regarding the need for the adoption of a “National Policy for Rare Diseases” in Brazil ^15^, and in January 2014 a Rare Diseases National Attention Policy was established ( MOH Ordinance No. 199).

This innovative policy adopts a broader perspective regarding rare diseases, taking into consideration the established Brazilian National Humanization Policy (PNH) and, thus, the following needs:

1) Comprehensive and multidisciplinary healthcare for people with rare diseases;
2) Standards for the qualification of Specialized Care Services and Reference Services for Rare Diseases in the Brazilian public health system; and,
3) A definition for the scope of activity of Specialized Care Services and Reference Services for Rare Diseases in the Health System, in addition to establishing technical quality standards for proper performance of their duties in the context of the healthcare network. This also takes into account the assistance required by the health care managers in regulating the access, control and evaluation of assistance for people with rare diseases in the SUS.

Additionally, this new national policy establishes the guidelines for patients with rare diseases and the program financial funding for SUS. As defined by the policy, a rare disease in Brazil is one that affects up to 65 people in every 100,000 individuals, or 1.3 per 2,000 individuals. Brazil strives to achieve certain milestones through this law, which include the following: ensure the universality, completeness and fairness of health services for individuals with rare diseases, with a subsequent reduction in morbidity and mortality for patients with such diseases; establish set guidelines for people with rare diseases care in all the SUS care levels; provide and expand equal and regulated healthcare access for people with rare diseases; ensure that people with rare diseases have timely access to available diagnostic and therapeutic methods; and establish care for people with rare diseases.

After the establishment of the policy, a new public consultation took place to create a prioritized list of rare disease protocols, as established by the SUS ( Public Consultation No. 20). The National HTA commission (CONITEC) had an intensive scientific collaboration on this process and will be responsible for the creation of 12 new guidelines in 2015 ( CONITEC Consultas Públicas). Currently, 35 treatments for rare diseases are covered by the SUS. It is important to remember that, in Brazil, the public health system is a right for all 202 million citizens living in the country.

### Countries currently without a definition of rare diseases

***Peru.*** Currently, no definition for rare diseases exists in Peru. However, in 2011 the country passed legislation promoting treatment and a national strategy for rare diseases, which includes diagnosis, surveillance, prevention, care and rehabilitation for such conditions ^9, 10^.

## Specific recommendations for harmonization (based on suggestions of the international biotechnology industry organization [BIO])

The Biotechnology Industry Organization, a not-for-profit trade association representing more than 1,100 companies, academic centers and research institutions in over 30 countries globally, constructed a letter commenting on the Brazilian Ministry of Health's proposed Standards for Enabling Specialized Care Services and Reference Centers for Rare Diseases in the SUS and Guidelines for Integral Care for People with Rare Diseases in the SUS ^15^. These recommendations, while not universally appropriate for/specific to all LA countries, are useful as a benchmark for many developing nations. They, in conjunction with others from ICORD ( Acta Pediatra), are abstracted below:

- Adopt the EU definition of a rare disease;
- Identify a subset of rare diseases as “ultra-rare”— (Although there is no internationally agreed upon definition of an ultra-rare disease, the UK National Institute for Health and Care Excellence (NICE) defines an ultra-rare disease to be one that affects less than 1000 people in the UK. Other regional-specific definitions may be appropriate as well) ^16^.
- Do not categorize the diseases based on their etiology, but only by their prevalence, to reduce restrictions in patient access;
- Allow for different types of authorizations, (e.g., the EU has three approval pathways: full approval/authorization, conditional marketing authorization, and authorization under exceptional circumstances; the US has accelerated approval regulations based on surrogate endpoints followed by post-market confirmatory studies);
- Institute incentives for industry, such as tax credits (US) for R&D, fee waivers (US/EU), and market exclusivity (US/EU);
- Create early access/compassionate use programs, such as the French Temporary Use Authorization ^17^;
- Establish a federally-funded Coordination Center to act as a central information resource, a patient/researcher support system, and a promoter of public awareness;
- Establish a special technical subgroup for rare diseases within existing technology appraisal agencies so that the health technology assessment (HTA) processes (such as cost-effectiveness analysis/budgetary impact modeling) typically used are evaluated with an eye towards the unique challenges of rare diseases, e.g., very high cost/quality-adjusted life year (QALY) but low budgetary impact because of small patient populations. Include other factors besides cost, such as lack of alternative treatments, equity of care, fairness of process, and society’s willingness-to-pay;
- Adopt a comprehensive approach, including education, prevention, diagnosis, care and treatment; in addition, support social, basic and clinical research;
- Involve patient advocacy groups in identifying patients, making genetic testing available, providing resources for screening. Additionally, have the groups involved with informed consent and autonomous decision making, advisory groups and expert panels; and,
- Use a multi-stakeholder approach.

## Discussion

This article focused largely on legislation in order to address some of the inadequacies relevant to making drugs available for orphan diseases; of course, there are many mitigating factors surrounding the debate on market access. For example, as indicated in a recent European review of EU orphan drug policies ^18^, society’s values in regards to funding these drugs, which would never be considered as cost-effective as drugs for more prevalent diseases, need to be clarified in LA. Similarly, consideration needs to be given to the revision of pricing and reimbursement policies in LA. Certain questions remain unanswered: does there need to be a cap on profits that can be generated by a drug with an orphan designation before it “loses” that designation? What types of analyses, e.g., budgetary impact modeling, need to be undertaken to demonstrate the relative cost-effectiveness of these agents in their respective populations? In addition, it is suggested that determining and revealing specific research priorities, e.g., for specific orphan diseases as a group, would incentivize companies to undertake research in those areas.

Recommendations for the harmonization of orphan drug development in LA with that of developed nations include adopting the EU definition of rare disease, instituting incentives for industry, and establishing a special technical subgroup for rare diseases that considers the unique challenges of rare diseases, among other techniques. Implementation of some or all of these approaches, which have been used somewhat successfully in the US, EU and other developed countries, will play a large role in improving access of orphan drugs to patients in LA and other emerging markets with rare diseases. Considering the scarcity of resources and trade-offs involved for decisions pertaining to which rare diseases to address and how to address them, it is incumbent upon all stakeholders to promote harmonization and advances in legislation, regulation and market access schemes, with clear rules and incentives on orphan disease definitions and drug development. Indeed, Drummond and Towse suggested using a more collaborative manifesto amongst countries to address these issues, similar to the global business case employed by the pharmaceutical companies in development of these and other agents ^18^. This methodology, in conjunction with discussions about specific countries’ societal values and affordability issues, will help to address the needs of this special patient population.
