# Supplementary material for: The role of globalization in drug development and access to orphan drugs: orphan drug legislation in the US/EU and in Latin America
Source: F1000Res. 2015 Feb 27;4:57. [Version 1] doi: 10.12688/f1000research.4268.1 (PMC4367514; doi:10.12688/f1000research.4268.1)
Supplement: Peer review history

# Peer review: Referee response for version 1

**DOI:** 10.5256/f1000research.4569.r7869

**Published:** 2015-03-16

**Authors:** Wendy Lipworth

**Affiliations:** 1 Centre for Values, Ethics and the Law in Medicine, University of Sydney, Sydney, NSW, Australia

This article addresses an important issue: what countries can do to ensure that patients with rare diseases have access to safe, effective and affordable medicines. It is essentially a descriptive (rather than critical/analytic) article, that would primarily be of interest to those involved in orphan disease policymaking.

A few points for improvement:

1. It is not clear how the first part of title "The role of globalization in drug development and access to orphan drugs" relates to the article. The article is a description of orphan drug-related laws and policies in a series of countries, so it is not clear what is meant by "the role of globalisation"
2. The introduction could be organised better by discussing orphan drugs in general, and then giving the facts and figures regarding Latin America (rather than weaving the two together as is currently the case).
3. It would be helpful to know a bit more about the health systems in the countries discussed so that the descriptions of orphan drug provisions can be placed in context.
4. Finally, it would be helpful to know why the countries were divided according to the definition of rare disease that they have adopted. Was there some expectation that different definitions would lead to different policies and laws?

I have read this submission. I believe that I have an appropriate level of expertise to confirm that it is of an acceptable scientific standard.

# Peer review: Referee response for version 1

**DOI:** 10.5256/f1000research.4569.r7856

**Published:** 2015-03-03

**Authors:** John Forman

**Affiliations:** 1 New Zealand Organisation for Rare Disorders, Wellington, New Zealand

The authors add to the public domain valuable information about the legislative and policy steps in selected Latin American countries to deal with rare disorders. This is welcome in the context of the emerging globalisation of health polices modelled on the success of pioneering policies elsewhere, particularly in the United States, Japan and the European Union. The work describes actual policy initiatives in Latin America, and avoids the frequent limits of much discussion on this topic which often concentrates primarily on the US and EU experience and simply advocates greater adoption of such policies elsewhere. Here we see the real experience of the globalisation process in its impact in several countries in a specific region.

It is notable that the authors briefly describe some aspects of diagnosis, prevention, healthcare, and rehabilitation in their discussion. But the article begins and concludes with strong emphasis on discovery, regulation and reimbursement of novel drugs for rare diseases. While drug discovery and access are of great importance to rare disease patients and their families, so too is the rest of their journey through life in the health and disability system. This and future articles on the topic could be enhanced by greater commentary on how these other important matters are also addressed in various national plans.

The work highlights the need for similar investigation of rare disease policies in other world regions, to add to the global knowledge set.

I have read this submission. I believe that I have an appropriate level of expertise to confirm that it is of an acceptable scientific standard.
